# An image processing and analysis tool for identifying and analysing complex plant root systems in 3D soil using non-destructive analysis: Root1

**DOI:** 10.1371/journal.pone.0176433

**Published:** 2017-05-03

**Authors:** Richard J. Flavel, Chris N. Guppy, Sheikh M. R. Rabbi, Iain M. Young

**Affiliations:** 1 School of Environmental and Rural Science, University of New England, Armidale, New South Wales, Australia; 2 Sydney Institute of Agriculture, School of Life and Environmental Sciences, University of Sydney, Sydney, New South Wales, Australia; Institute for Resistance Research and Stress Tolerance, GERMANY

## Abstract

The objective of this study was to develop a flexible and free image processing and analysis solution, based on the Public Domain ImageJ platform, for the segmentation and analysis of complex biological plant root systems in soil from x-ray tomography 3D images. Contrasting root architectures from wheat, barley and chickpea root systems were grown in soil and scanned using a high resolution micro-tomography system. A macro (Root1) was developed that reliably identified with good to high accuracy complex root systems (10% overestimation for chickpea, 1% underestimation for wheat, 8% underestimation for barley) and provided analysis of root length and angle. In-built flexibility allowed the user interaction to (a) amend any aspect of the macro to account for specific user preferences, and (b) take account of computational limitations of the platform. The platform is free, flexible and accurate in analysing root system metrics.

## Introduction

Non-destructive observation and measurement of plant root traits in soil represents an important goal for many plant and soil scientists [1]. Ideally, this would be achieved in the natural field environment. However, past experiences of excavating soil pits and exposing a vertical soil surface under field conditions, and then measuring a small fraction of exposed plant roots, have shown that such methodology is sub-optimal [2,3] with only a small portion of the total root system observable and no 3D spatial information retained.

Whilst interesting and elegant approaches to monitoring and measuring root growth are developing in soilless transparent media [2,4] omission of soil from such experiments and assays is also sub-optimal as it excludes the soil-root interface which is the main reactive surface in plant development in terms of water/nutrient uptake and root development, and is important for all soil biological processes [1,5,6].

Research in non-destructively observing soil and plant development has increased significantly over the past 40 years. From the early 1970’s, when the first tomography scan was attempted in soil systems, a yearly doubling of publications in relation to plant root and soil investigations (Google Scholar accessed 17^th^ June 2016) has occurred. As interest in fast, high throughput, root phenotyping increases [7], the advantages of high speed, accurate, segmentation and analysis protocols revealing root architecture in 3D soil becomes more necessary and advantageous. What is clear is that no current non-destructive technique will allow us to observe and measure all the roots of a single plant [8,9]. However, greater capture of significant volumes of the root system, at higher spatial resolutions, is now possible. This necessitates the development of methodologies that are capable of dealing with large and complex data sets, both from an image manipulation and analysis viewpoint.

The majority of the literature using x-ray micro-computed tomography (μCT) for root imaging has been in validating the technique [10] and the strongest correlations with destructive measures of root metrics have been obtained using methods with a high degree of user interaction [11, 12, 13]. Identifying and measuring roots in soil even with non-destructive analysis techniques remains a difficult task. Several authors [9, 13, 14, 15] have explored the use of μCT for translating the form of soil and soil-plant characteristics to functional traits. With caveats, these approaches have proven successful in developing experimental and software approaches to understand soil-plant interactions in 3D within a more natural heterogeneous habitat. Examining young, and geometrically simple, root architectures Gregory *et al*. [16] captured 90% of 7-day-old, short, wheat. Perret *et al*. [17] retreived approximately 87% of the total root segments, and 78% of the total root length in chickpea. Recently, Paya *et al*. [18] retreived high quality and very accurate root data from tree roots. However, polystyrene balls rather than soil was used as a growing medium. Tracy et al. [19] observed relatively low correlations between μCT observed roots and those harvested destructively (r^2^ = 0.53); whilst more recently Lazorenic et al. [20], examining the impact of Al toxicity on wheat root architecture, found that μCT identified only c.75% of the roots observed during destructive harvesting.

A key on-going requirement is both an accurate segmentation and measurement of roots and a significant automation of all the key processes alongside the development of free flexible software that allows changes to be made by the user, to take account of the variable experimental protocols and the wide range in plants and soils used. It is clear that no single processing solution is applicable to all experimental conditions.

This work aims to provide image manipulation and analysis protocols which will be freely available on the ImageJ website. A key aspect of the proposed protocols is the ability to automate many of the important image-processing steps previously only manageable by manual implementation. Also, users are able to amend any portion of the proposed macro to suit their experimental protocols. We introduce a semi-automated protocol that permits the segmentation of complex cereal root systems and more simple tap root systems. Finally, it should be noted that our proposed protocols are not restricted to μCT data, and will in theory deal with any 3D volume no matter the technology (MRI, Neutron etc) used to capture the data, with only slight modifications. The developed macro is called **Root**1.

## Materials and methods

### Growth and scanning parameters and protocols

Chickpea, wheat and barley varieties were chosen as they represent a range of commercially important crops with geometricaly complex, yet significantly different, root architectures providing a range of challenges to overcome for μCT scanning and subsequent image processing: one a taproot with relatively large root diameters, but with a less variable architecture, compared to highly complex and adaptable wheat and barley root architectures with relatively small diameter roots. This paper focuses on the post processing of μCT images, and correspondingly important growth and scan parameters are included for reference purposes only. All plants were grown in PVC pots (55 mm diameter, 2 mm wall thickness, 260 mm high). Information is available on typical root architectures of various crops [21, 22]. The latter research observes wheat varieties exhibiting lower root length and higher root tips compared to barley (heirloom and commercial) cultivars.

### Chickpea

Two desi chickpea (*Cicer arietinum*) varieties, Yorker and D08260, with three replicates each, were sown into a Vertosol soil. The soil was packed to a uniform density of 1.1 Mg M^-3^ (5 layers individually packed to the required density to decrease variability—columns that are not packed as uniformly as possible, with boundaries that have highly connected pores at the resolution limit may cause some false identification of ‘root’ voxels). Water was maintained to a volumetric water content of 28% w/w daily (volumetric water content at field capacity 40%). Plants were grown for 14 days, after which time the entire root zone was scanned using the GE-Phoenix V|tome|xs with an isotropic voxel side length of 65.0 μm with parameters (130 kV, 210 μA, 0.125 mm Cu filter, 1800 projections using constant rotation CT method, 200 ms acquisition time). Four adjoining scans of the root system (top to bottom) were acquired.

### Wheat

A single wheat (*Triticum aestivum* L.) Cv. Sunvale plant was grown for a period of 28 days in a Ferrosol soil [23] under conditions described previously [11]. These conditions aimed to quantify root responses to banded phosphorus. The plant was scanned using the GE-Phoenix V|tome|xs with an isotropic voxel side length of 27.0 μm (x-ray tube parameters: 160 kV, 200 μA, 0.125 mm Cu filter, 3600 projection angles using constant rotation CT method, 200 ms acquisition time). In total, 4 adjacent scans from a single plant were processed. Image processing was carried out as detailed below using FIJI, ImageJ version 2.0.0.0-rc-15/1.49k, Java 1.6.0_65.

### Barley

A single barley plant (Gairdner) was grown for a period of 20 days in a Ferrosol [23], at a P rate of 150 mg kg^-1^ and a N rate of 65 mg kg^-1^. The plant was scanned twice: once at high voxel resolution (27 μm) and then at a lower resolution (57μm) with x-ray tube parameters and image processing identical to the wheat scanning (above). Scanning at different resolutions was carried out to make a comparison of the effect of spatial resolution scan on the recovery of observed root system. Barley was chosen as another test for the image processing for a different complex root architecture.

To avoid operator bias in any of the processes used, two independent operators scanned and physically extracted the wheat and barley root systems, the chickpea roots were scanned by the operator of the wheat root scans.

### Manual root measurements

Following μCT scanning, all soil was removed from the pots and washed with water over a 500 μm mesh to extract the root systems. Roots were then preserved in 50% ethanol solution and stored at 4°C. The washed roots were imaged using an Epson V700 scanner at 600 dpi (i.e. pixel side length 42 μm) and analysed by WinRhizo v. 2009c software (Regent Instruments Inc., Quebec, Canada).

## Micro-computed tomography image analysis steps

Our aim was to use publically available ImageJ algorithms, along with relatively simple and accessible steps that we have deployed that improve segmentation. These are described below.

### Alignment of histograms and volume stitching: Normalizing the images

**Fig 1:**
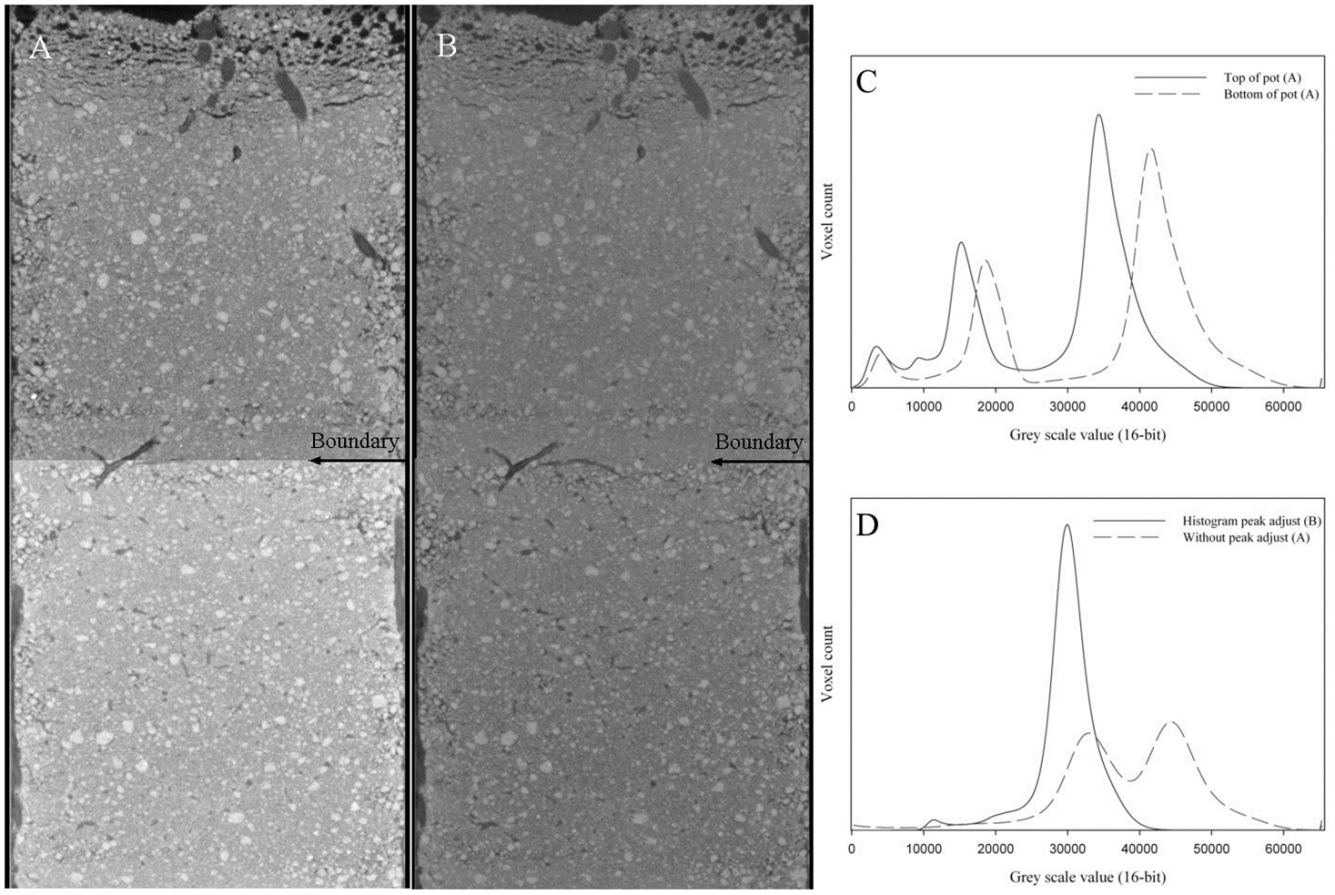
Influence of drifting grey values in individual scans on final combined volume grey value continuity. (A) A single sample scanned in two parts (consistent scan parameters) with subtle compositional changes causing different grey values to be assigned. When digitally ‘stitched’ together, the resulting volume produces discontinuous grey values. (B) The same sample having applied the *‘Align Peaks’* method to produce uniform grey scale assignments across the two volumes. (C) Grey scale histograms of the individual top and bottom scans of [A] displaying the shift in grey value assignments and the relative similarity of the histograms. (D) The grey value histogram of the stitched images.

The histograms of successive scans were aligned using a peak matching method. The peak matching method identifies a localised peak of the histogram (the soil peak in our samples), and aligns these to a coincident grey value set by the user. Initially the 32-bit tomographs are scaled to a defined grey value range. The data is then converted back to 16-bit depth to reduce image size and optimize image contrast. This process controls the range of grey values observed between sequential scans and provides more consistent grey values between scans [13]. It is a simple, computationally efficient and automated method. As far as we are aware, no other published paper on μCT and root analysis, implements such a strategy. Fig 1 illustrates the advantages of this process in ensuring appropriate comparisons between scans. Without this step any subsequent segmentation between scans would be significantly compromised, with potentially substantial errors introduced.

It should be noted that the peak matching method is not suited to all applications as it assumes that the variance observed in the histogram is relatively consistent between scans. For the data presented in this paper, this was the case. However, in circumstances where the range of a substance’s attenuation values change (e.g. where there are extreme changes in sample composition, for instance in a soil core with abrupt changes in bulk or particle density between horizons) this method is less applicable. In such cases, histogram equalisation (for uni-modal data) and contrast enhanced adaptive histogram equalisation (for multi-modal data) are more appropriate. These protocols are available as ImageJ plugins if required. However, this may increase computational demands [24].

Four adjoining scans of the whole rooting zone were stitched to provide a continuous and seamless volume. This was achieved by ensuring that each scan had a small region of overlap at the boundary. Duplicate slices are automatically identified and removed, concatenating the remaining slices into a single stack.

### Finding the roots

**Fig 2:**
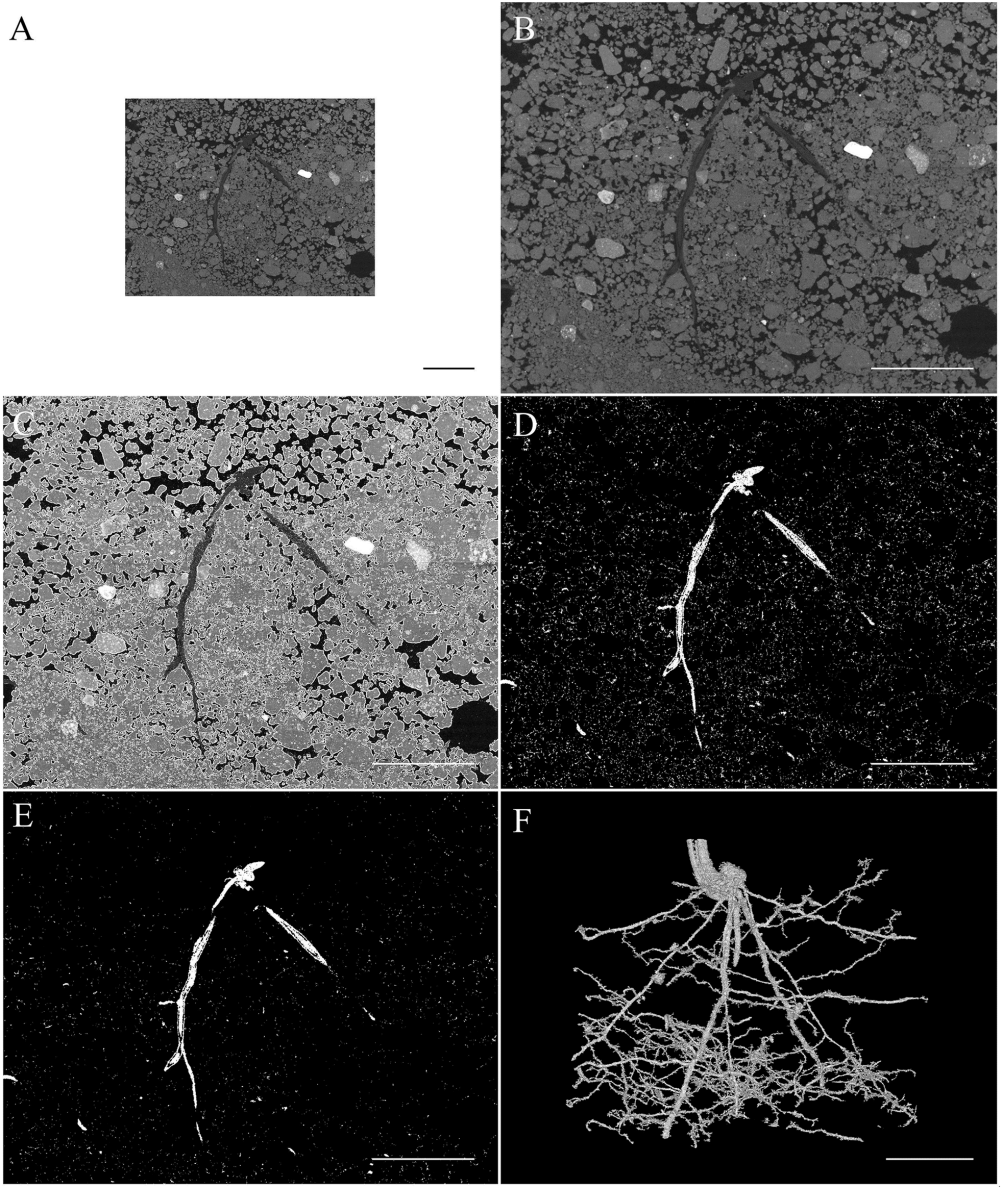
Processing sequence of operations for segmentation of root system from CT scans. For all images, the scale bar represents 10mm and the images depicted represent a small subsection of the total volume scanned (A) A single cross-section slice of the tomograph following image stitching of two sequential scans. The roots are centrally located and represented by mid grey values. (B) The same slice of the tomograph following resizing, where each voxel is expanded by a factor of two in each direction. (C) Single cross-section slice of tomograph following the identification and removal of partial volume effect pixels using a sobel filter. Isolated voxels are indicated in white tones. (D) Cross-section slice of tomograph after threshold is applied to isolate the root system. ‘Root’ voxels are represented by white. (E) Thresholded root system (white) following median and erosion filters to remove fine pore voxels connecting roots to non-root volumes. (F) 3D volume of the root system following selection using a connectivity algorithm.

Now the images have been normalized, the next stage was to identify the roots. The use of medical CT systems in human health investigations has the advantage in that a fine detail map of the human body is available in which medics seek abnormalities (e.g. tumors, bone fractures etc). In soil, we are looking to find ‘soft’ biological roots in hard (organomineral), and/or empty (soil pores), and/or water-filled pore volumes all within a highly heterogeneous maze. This is several orders of magnitude more difficult. The following steps outline the processes we employ to manipulate the images with the aim of optimising the segmentation of roots from soil and pores. Essentially, we use a balance of increasing sample size, filtering, thresholding, and erosion techniques. Fig 2 provides example outputs of each step to provide a visual summary of the protocol.

### Artifically increasing volume size

Since two of the following procedures in this method involve the spatial separation of non-root features and the isolation of the root structure based on connectivity, it was necessary to increase the number of voxels contributing to the root diameter so that erosion of the root exterior did not cause root fragmentation. Thus, the dataset was resized to increase the number of voxels in every direction by a factor of two (thus the volume was represented by 8 times as many voxels). This preserved connectivity for extracting the root system by its connectivity later. The new voxel values are assigned by bilinear interpolation, which also has a smoothing effect on the grey values. This process was performed using the ImageJ ‘Size’ tool and Fig 2A and 2B provide typical outputs for these steps. It also increases the file size and processing time. However, as the process is automated, this only represents computational time.

### Removal of partial volume effect

The partial volume effect (PVE) in CT volumes is caused by the discretisation of a continuous spatial volume (the sample) into defined voxels. Basically, one voxel can contain two, or more phases (say a pore and soil component). The resulting grey value then represents an average of these phases [25,26]. This is especially important to take account of in relation to root systems which can be thought of as a bundle of biological edges positioned at the sides of pores and/or solids and thus more than most objects are highly prone to PVE errors. This is problematic as the voxels at the air:soil interface are often assigned the same grey value range as the root system.

The second step in this method is to identify the interface of the air soil phase boundary by its steep gradient. A 2D Sobel [27] edge detection filter (implemented as the ImageJ ‘Find Edges’ tool) was used resulting in the square root of the sums of squares of two directional 3x3 convolution kernels. The resultant image is proportional to the probability of that voxel being at the air soil interface. With appropriate thresholding, a mask can be created which is used to exclude the partial volume effect while leaving regions of more subtle grey value gradients, such that occur at the air:root or root:soil interfaces. Fig 2C provides a typical output of this procedure.

The abundance of partial volume effect (PVE) voxels in the grey value range common to root tissue clearly makes global thresholding as a method of extracting roots inadequate [16, 15]. It also contributes to the change in grey values assigned to roots with soil depth and diameter [12, 28]. This is in part an explanation for the need to employ semi-automated local adaptive thresholding to identify complex root system structures, as thresholds are manually determined appropriate for the local region [11, 12, 19, 29]. We found that the combination of a 2D Sobel [27] edge detection filter with the FIJI automatic threshold (based on isodata iterative intermeans [30]) is successful at identifying the PVE voxels at the soil:air boundary. It should be note that while this approach successfully removes the voxels surrounding pores sufficiently greater than the voxel size, it is not effective on pores smaller than approximately 3 times the resolution. Galkovskyi et al. [31] provide an excellent analysis of various thresholding techniques on root system measurements.

### Thresholding and filtering

Our aim is to identify the voxels belonging to relatively fine and coarse roots from the various grey scales offered within each scan. Also, as there is a lower spatial threshold below which we cannot identify roots (as determined by the scan parameters), we binned those scales below the limit, making the identification of root grey scales less complicated. Global thresholds can be problematic in identifying root systems as different parts of the root system can change in attenuation depending on root diameter and root tissue type (eg. if aerenchyma is present) [32]. However, now that much of the partial volume effect is removed from consideration, a broader threshold range was then used to include both coarse and fine roots. Here we employed a bilevel threshold (predetermined by the user) to extract the remaining voxels that fall within the ‘root’ grey value range. This step did not exclude the small pore space previously identified as missed by the edge detection mask. Fig 2D provides a typical output for this procedure.

Much of the binary image extracted by the bilevel threshold represented either root or fine pore space which was connected to the root, or other pores by a fine network of voxels. Many of these structures were removed using a 3D median filter operating on a 3x3x3 neighbor matrix (ImageJ plugin “Median (3D)”). This had the effect of removing small scale structures, small ‘tabs’ left on the root system from nearby pores and cleaning the image of fine scale noise while large structures remain intact. This is one of the steps that justifies initially resizing the image as fine roots are now represented by a larger number of voxels and as such are more impervious to this kind of filter.

### 3D erosion

An optional 3D erosion step is included, where a user selectable number of erosion operations can be performed using a kernel with a diameter of 3 voxels (ImageJ plugin “Erode (3D)”). This filter is suitable for eroding small structures connecting fine pore space to the surface of the root (Fig 2E). This also erodes a voxel from the surface of the ‘root’ which can disjoin the finest root fraction or tenuously connected regions of root. However, the filter erodes the equivalent of sub-voxel steps (at the original resolution) since the volume was resized and therefore improves the detection limit for the smallest diameter fraction of the root system. This step is not always required but is particularly important where experimental protocol has resulted in a high proportion of connected pore structures that are less than 3 times the voxel resolution in diameter. In Fig 2E this stage is represented by a significant decrease in white pixel noise.

### Optical extraction of roots

**Fig 3:**
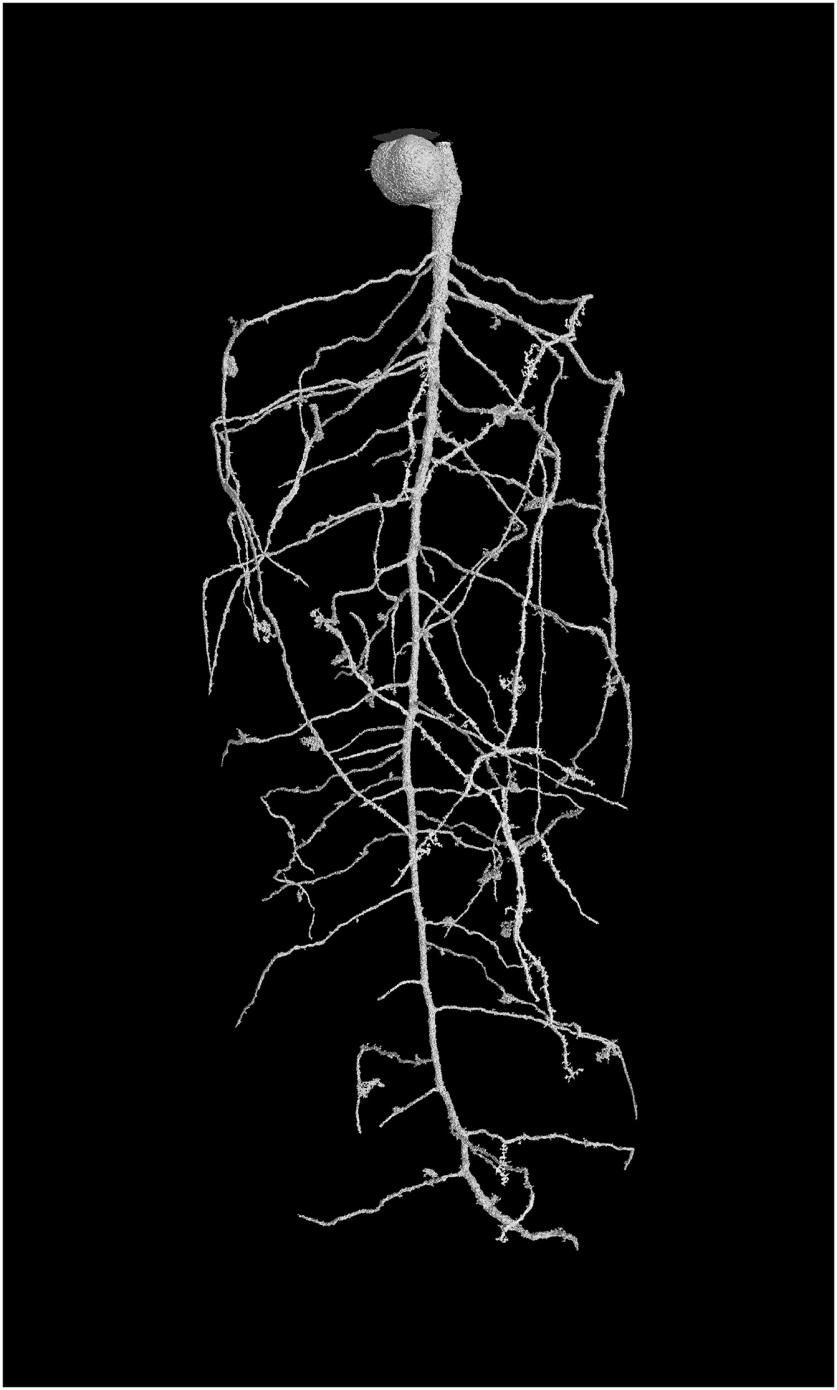
Example of segmentation output 4 vertically adjoining scans of chickpea (*Cicer arietinum*) var. Yorker root system 14 DAS. Automated segmentation was used to extract the root system from CT scans using a single user determined seed point. Rendering performed using VG studiomax 2.0.

A binary image is now created containing a connected root system interspersed with disconnected pore space. In this state, it is difficult to extract meaningful measures of the root system, its distribution or architecture. Having addressed the partial volume effect and the digital connection of the root system to other scan features, which significantly reduces contrast errors, it is now easier to extract the root system from a predetermined user defined seed point which provides a useful method of identifying and extracting only the root system. There are a number of connected region plugins in the ImageJ platform that are effective for small files but have computational and memory limitations when processing larger volumes. Small files such as that represented in the chickpea example (Fig 3) are managed totally within Root1. Due to memory restrictions in using ImageJ (2.12x10^9 voxels in size—which equates to about 2120 slices at 1000x1000) the larger wheat column data was exported into VGStudioMax version 2.0 (Volume Graphics Gmbh, Germany). This is small limitation of our current macro; whilst there are protocols that could be employed in using ImageJ for larger files, none would be close to the speed of reconstruction offered by the readily available software available in the many CT platforms, that always come bundled with every CT system, and that are optimized for processing large 3D datasets without having to load the entire volume into memory. Additionally, as the Root1 macro is Public Domain and highly flexible, any user will be able to add new protocols as required by individual labs and use any computer facility with fast processors and large memory allocations, which are becoming the norm in most labs.

**Fig 4:**
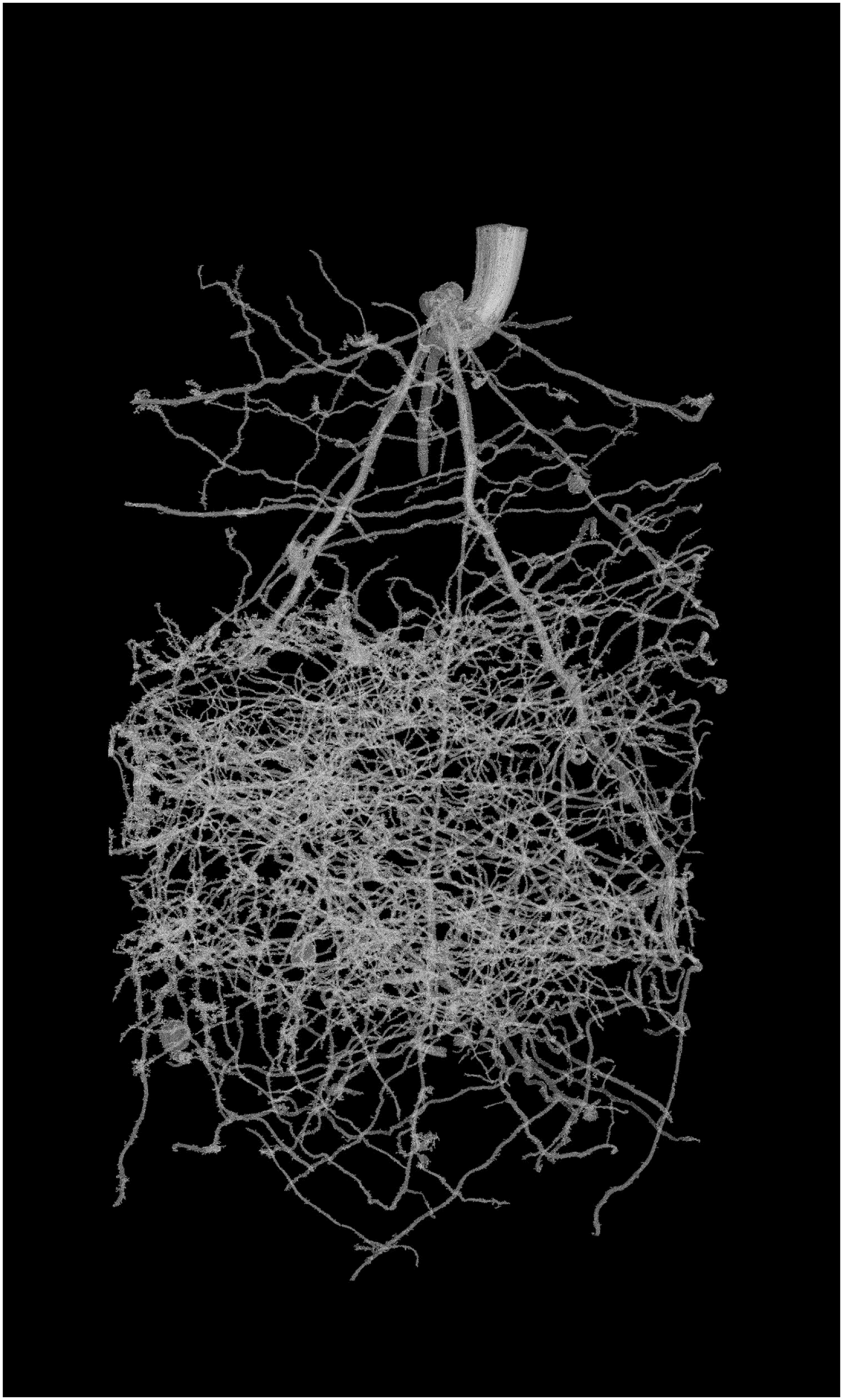
Example of segmentation output 4 vertically adjoining scans of Wheat (*Triticum aestivum* L) var. Sunvale root system 28 days. Automated segmentation was used to extract the root system from CT scans using a single user determined seed point. Rendering performed using VG studiomax 2.0. The raw root volume data was taken from the Flavel et al., 2014 data set.

**Fig 5:**
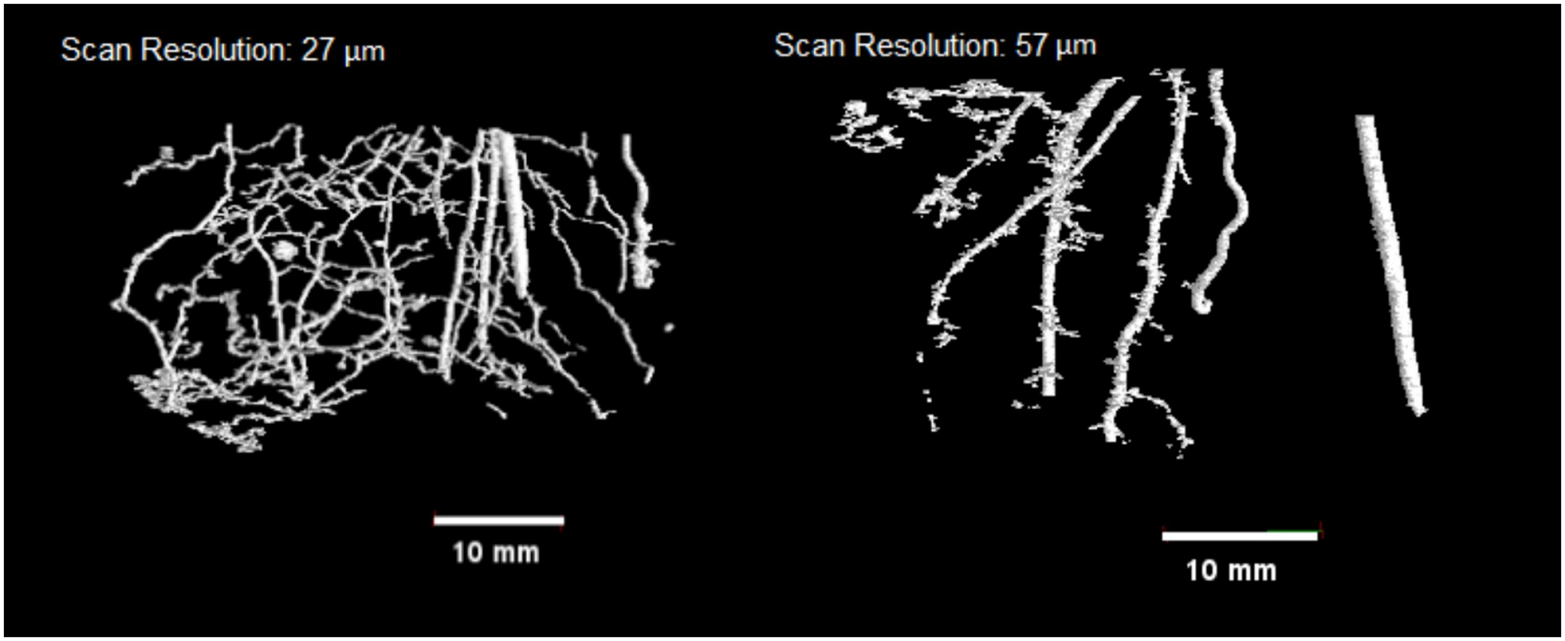
Example of segmentation of the 20 DAS barley root system. A. High resolution (voxel size 27μm) and B. Low resolution (voxel size 57μm).

The root system is selected with a single ‘click’ using the Region Growing tool in VG Studio max, which is based on local adaptive thresholding. The threshold set for this operation becomes arbitrary as the dataset is now binary. If required for samples with significant pot edge effects, or where disconnected structures are of interest, the volume can be explored and the user can manually identify disconnected root fragments. However, the purpose of the above processing is to limit user interaction to a single click. The benefit of using a memory optimized program such as VGStudioMax is that applying a connectivity process to the whole volume simultaneously means plagiotropic branches can be extracted without employing an iterative process. Once identified the root system can be exported for architectural analysis and the volume can be re-binned to its original voxel dimensions (using the ImageJ ‘Bin’ tool with ‘maximum’ setting) maintaining root structure while reducing file size (Fig 2F). Examples of the extracted root systems are provided below for the chickpea root system (Fig 3), the wheat root system (Fig 4) and the barley root system (Fig 5). It is clear from each image that the procedures used retain the essential elements of each quite different root architecture.

### Analysis of extracted root volume

Root length was targeted as a highly descriptive and verifiable root morphological trait that was reflective of conventional root methods and useful for characterising root system architecture. When applied to segmented root volumes, skeletonising algorithms remove sequential exterior layers of root voxels until the centroid of the root is identified. With calibrated images, voxel counting of the skeleton can be used to determine the length of roots or root branches [33, 34]. The ImageJ-FIJI plug-ins “Skeletonize (2D/3D)” and “Analyze Skeleton (2D/3D)” (an implementation of [35, 36]) were used to generate a list of all branch lengths in the root volume. These branch lengths represented the skeletonised length along the root path between two branches, or in terminal branches between the branch and the tip of the root.

The major benefit of including the root skeleton in analysis is its capacity to analyse the spatial distribution of root architecture independent of root diameter. It is also necessary for identifying branching structures, from which branching or growth angles can be measured. The mapping of root length density is relevant for questions pertaining to plant root development and the exploration of soil regions. It is similar but not congruent to the 3D distance maps presented by [12] which quantifies the distance of the soil space relative to the nearest root surface. This is highly relevant to the uptake of resources in the root zone. In this manner the spatial distribution of root architectural characteristics length and branching can be measured in 3D and at high resolution.

**Fig 6:**
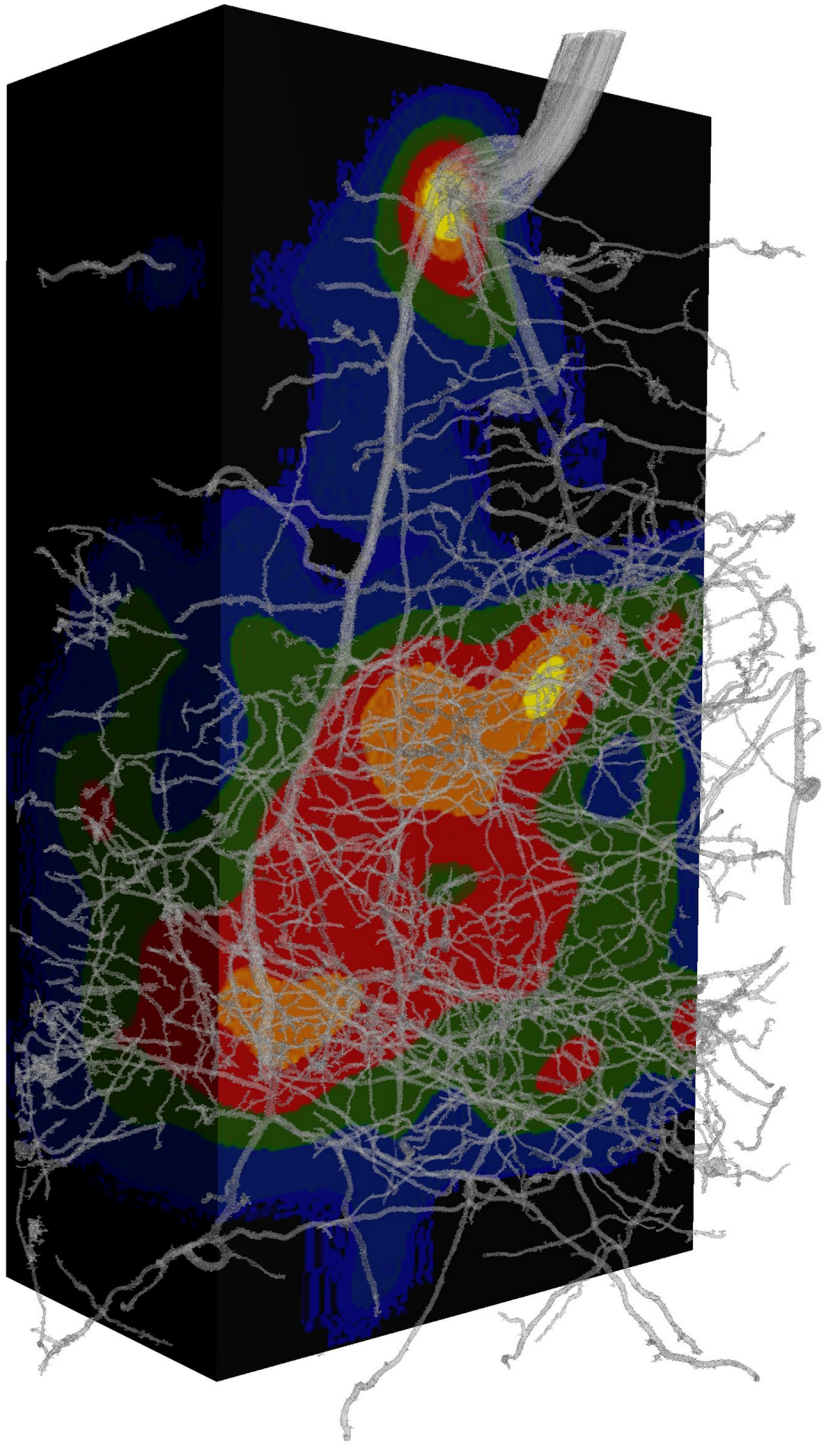
Root length density (RLD) map of the of the wheat root system presented in Fig 3. Cool colours represent low local RLD while warm colours indicate high levels of local RLD. High RLD in lower half of the volume are in response to a local phosphorus band. Rendering of 3D volume performed using VG Studio 2.0.

An automated macro was developed to measure length, root length density, branching and branching density based on the skeleton structure. The “Analyze skeleton” function is used to classify skeleton voxels with 1, 2 and 3 nearest neighbours, relating to tips, root length and root branch sites respectively. The classified root skeleton voxels can be counted in a slice-by-slice manner, from which the total root length and root length density as well as branching density can be calculated relative to depth of the pot. While this method is relevant for experimental designs where root metrics are relative to depth [14] this technique can be extended to quantify the 3D distributions of root length density which is more relevant to plant responses to the localised spatial heterogeneity of the edaphic environment (Fig 6).

**Fig 7:**
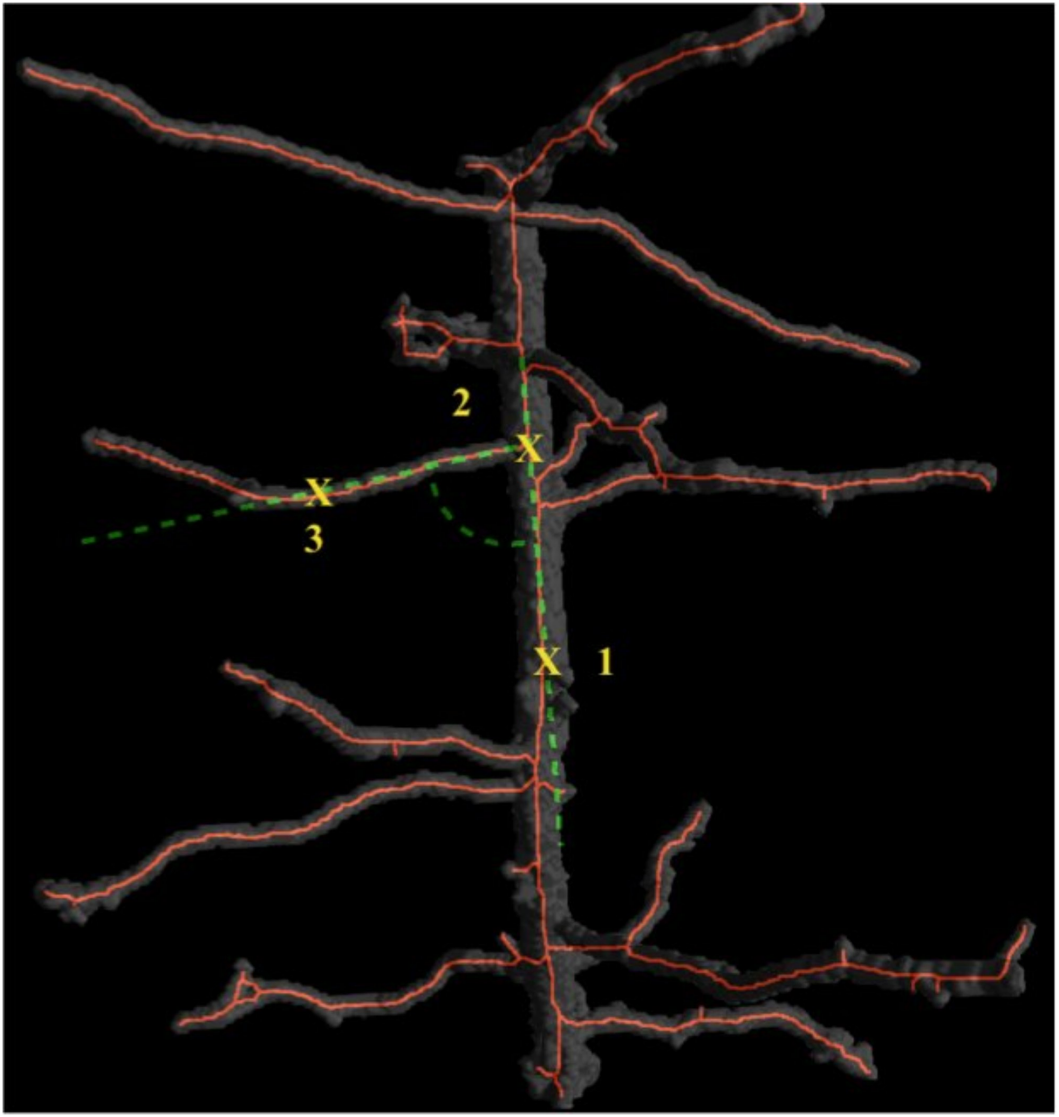
Three dimensional visualisation of the root branching angle tool. Root branch angle can be measured using thee points; along the parent root (1), at the branch vertex (2) and along the branch root (3). 3D root skeleton (red) is superimposed on the root segmentation (grey) and the angle calculated is indicated in green (broken line).

Finally, a tool was designed to extract branch angle data from tomographs. The method superimposes the skeletonised information (with branching points labelled) onto the root volume stack. The user is able to scan through the images and with three ‘clicks’ identify the main root, vertex (branch point) and branch direction (Fig 7). The macro then records the angle between the three voxels. The resultant angle (between the bottom of the root and the downstream side of the parent) was selected as the standard measure as this is the angle commonly used in root system branch angle modelling [37, 38, 39].

## Results

Typically, for our Chickpea samples the scan times were around 25 minutes with a processing time of c.110 minutes. For the more extensive high resolution wheat and barley systems, the scan times were 50 minutes, with a processing time just over 186 minutes.

The processing time of a single sample using this method was directly proportional to a) the scan parameters of the samples (published scan times range from 6 min to 12 hours although times are rapidly becoming faster), b) the size of the dataset (how many sequential scans and scan dimensions), and c) the capacity of the computing facilities. Using a desktop computer (Mac Pro (late 2013) with 3.5Ghz 6 core Intel Xeon E5 processor, 64GB RAM running OSX 10.9.5), files in the order of 1000x1000x3000 can be processed in approximately 2 hours of compute time with additional human intervention time for setup and identifying the root of approximately 15 mins. This method is rapid when compared to the more labor intensive methods reported previously reported [11, 12, 19] which require considerable human interaction time (in some cases 10+ hours).

Careful examination of the extracted portion of the wheat root system as compared to the original scan indicated that this process extracted roots with diameters 4–5 times the voxel size. In the case of the wheat and barley root systems (voxel size 27.0 x 27.0 x 27.0 μm) we were able to extract roots with diameters at or greater than 100 μm in diameter. In the case of chickpea, roots at, or greater, than 260 μm in diameter were extracted.

Comparing the manually extracted roots to the μCT extracted roots, the chickpea root volumes had a coefficient of determination (R^2^) of 0.96. The regression equation Lc = 0.87Lw–26.2 (where Lc represents root length as determined by CT and Lw indicates destructive analysis) indicated a 10.8% underestimation of root length by CT that is comparable with the best results from previous studies [11, 12, 14], but determined with significantly greater automation and speed and accuracy. The wheat root system produced a 1% overestimate of root length as compared to destructive harvest (902.73 cm compared to 894.26 cm for CT and destructive methods, respectively). The high resolution barley root system (Fig 5) produced an overestimation of c.8% compared to destructive harvest (53 cm compared to 49 cm for CT and destructive methods, respectively). For the barley low resolution scanned roots, the CT observed root length was measured as 23 cm, a significant underestimation from the destructive measure.

## Discussion

In this study we sought to provide a freely available protocol to identify and measure root architectures and traits within 3D soil. The developed Root1 macro will be freely available on the ImageJ public domain website with the relevant supporting documentation. We also set out to present a rationale for each step.

**Fig 8:**
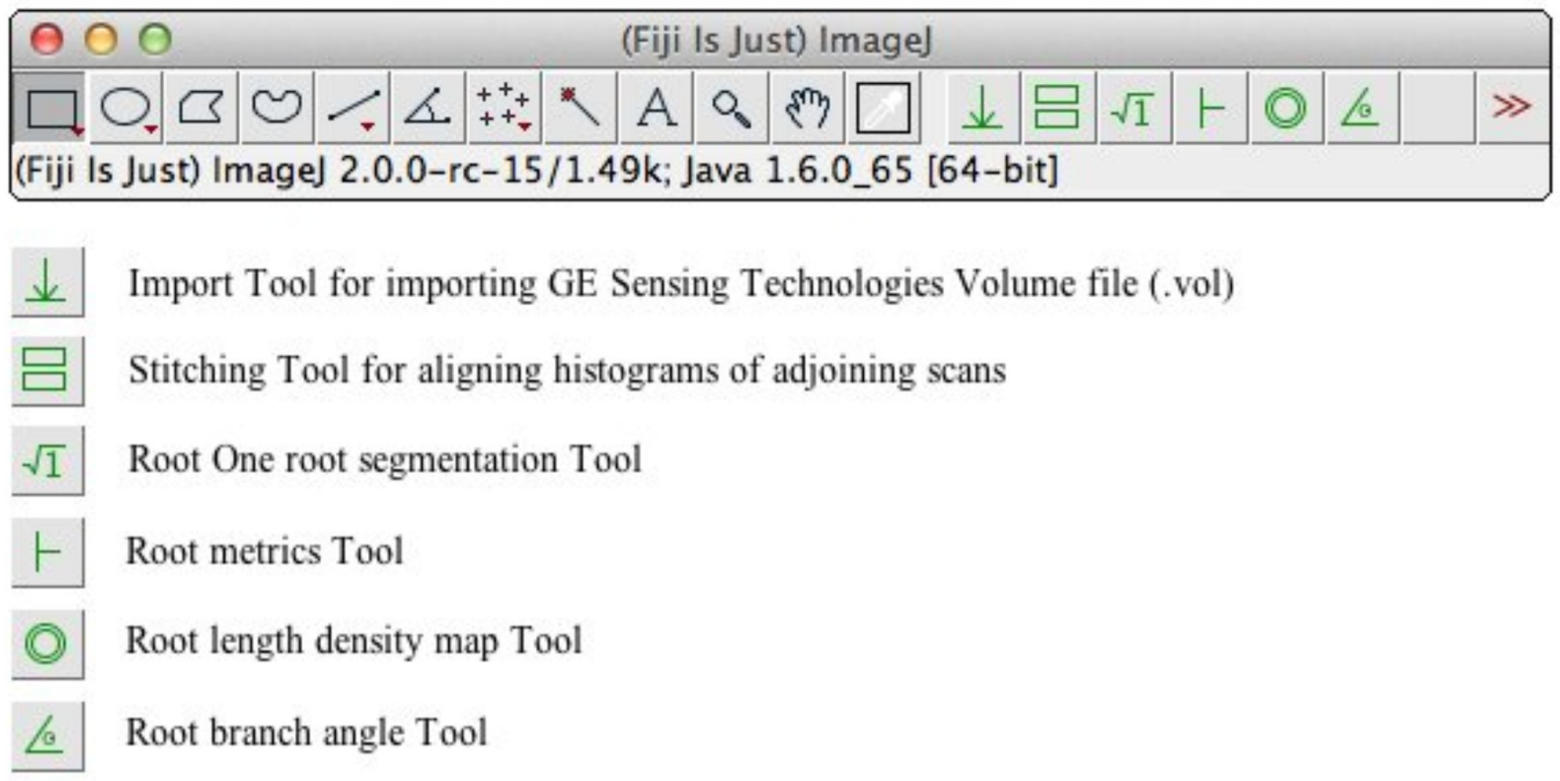
ImageJ macros are accessible as a toolbar for easy access but can be readily customized for different experimental protocols. The custom tools (identified from the default suite by the green color symbols) are (left to right) ‘Import tool’ for directly importing GE Sensing Technologies volume files into ImageJ; ‘Stitching tool’ which uses peak fitting to merge sequential scans for more consistent values; ‘rootone’ segmentation tool for extracting roots and export for connectivity filter; ‘Root Metrics’ tool which reduces volume size and measures skeleton characteristics; ‘Root length density map’ tool and root branch angle macro for manually measuring branch angle.

Our proposed platform is readily customizable so that segmentation protocols can be adjusted according to circumstance. The ImageJ platform with its many scripting options provided such a format [39]. Our approach seeks to tie together the significant amount of man-hours collectively invested in algorithm development of researchers and computer scientists to provide a solution to this problem with rapid development time. The macro language developed for ImageJ provides an ideal customisable format with user interface tool bar options (Fig 8).

Extraction of root tissue from the μCT data of both fibrous and taproot species provided good correlation with destructive harvests and analysis using WinRhizo. The wheat root system produced a small 1% overestimate of root length as compared to destructive harvest which is likely due to the inclusion of some soil organic components extracted with root tissue not included in destructive harvest (e.g. a small weed root was identified in the extracted cereal root volume).

The reality is that scan times and processing times can be reduced simply by scanning at lower resolutions and/or using less x-ray projections; all resulting in capturing less roots. As previously mentioned, no non-destructive system at present (i.e. x-rays gamma, neutron) is able to capture all roots that are present in soil. In our samples, where we capture roots at high spatial resolutions, the time allocation is reasonable. Naturally, both processes can be batched and only involve an operator at the initiation stage of each scan and the first processing step for each sample cohort–although we have flexibility inherent in our macro which allows operator intervention where deemed necessary. The biggest time constraint on most scans of root systems is ‘cleaning up’ the images, which our macro deals with in the preprocessing steps as described.

The low resolution scan from the barley plant provides an exemplar for choosing the correct spatial resolution for scanning. The reality is that μCT systems are limited in what they can observe at specific spatial resolutions within relatively large volumes. Low resolution scans of the barely plants captured only large roots, and significant noise within the 3D volume. Whilst the scan was considerably faster, the reality is that ultimately it does not serve any useful purpose in relation to observing and measuring the majority of the root system. Thus, it is not a limit of the proposed image processing as developed in this paper, or indeed any other processing system. Rather, it demonstrates a limitation of the use of all current cone beam tomography systems. Scans times are becoming significantly faster, producing larger volumes of 3D datasets, that ultimately have to be processed and analysed. However, the key is aligning the spatial resolution with the object of interest.

## Conclusions

The use of μCT for routine analysis of below ground characteristics was initially hindered by resolution and acquisition limitations. Recent challenges relate to functional segmentation algorithms [14, 15]. Both constraints have lessened somewhat to allow experimental measurement of root systems using CT to uncover the hidden half of the plant and the plant- soil dynamics in both time and space. Root system extraction from CT scans will always be dominated by the resolution limit of the technique. However, with appropriate selection of scan parameters root system features can be reliably observed. Rather than attempting to generate a single method for all situations, we have adopted a pragmatic approach to root system segmentation, where the algorithm can be adjusted relative to the nature of the soil and root system to reliably estimate relevant root metrics for breeding and research objectives.

The chickpea, barley and wheat images presented in this paper demonstrate that a large fraction of the root system can be accurately identified and measured for realtively simple and more complex root architectures, in soil. The accessibility and flexibility of the macro will provide a platform for many researchers to now take advantage of the developing non-destructive analysis techniques for root-soil interactions. The Root1 macro developed in this study will be made freely available in the ImageJ plugin page (http://imagej.nih.gov/ij/plugins/index.html).

Coding for a parallel processor would only significantly increase the processing times. As yet, this has not been acheived, but warrants further investigation, as the main issue for any nondestructive technique is the handling, processing and storing of big data sets.

Our protocol also allows capture of root architecture data, in 3D soil, that can then be used by a range of modelling programs, looking at a wide range of fundamental questions such as water utilization, solute uptake, and rhizosphere function.
